# Determination of the Phenolic Content in Iranian Trehala Manna and Evaluation of Their Antioxidant Effects

**DOI:** 10.1155/2021/8570162

**Published:** 2021-08-31

**Authors:** Farzaneh Darikvand, Mehrdad Ghavami, Masoud Honarvar

**Affiliations:** Department of Food Science and Technology, Science and Research Branch, Islamic Azad University, Tehran, Iran

## Abstract

One of the most challenging issues in the food and pharmaceutical industries is finding effective and safe antioxidants from natural resources compared to their synthetic compounds, which have side effects. In this regard, Trehala manna was considered a great antioxidant source categorized as the major type of manna produced naturally and by the *Echinops* plant in response to insect activity. In this study, the antioxidant activity and phenolic content of the numerous Trehala manna in *Echinops* sp. have been investigated. Different methods of radical scavenging activity comprising 2,2′-azino-bis (3-ethylbenzothiazoline-6-sulphonic acid) (ABTS) and 2, 2-diphenyl-1-picrylhydrazyl (DPPH) have been investigated to evaluate antioxidant activity. The phenolic contents were measured by Folin–Ciocalteu and standard phytochemical methods. Quantitative and qualitative amounts of phenolic content, including caffeic, ferulic, coumaric, syringic, and hydroxybenzoic acids, were quantified by high-performance liquid chromatography (HPLC). The results indicated the significant changes in the amounts of phenolics and the antioxidant properties in Trehala manna samples, based on the place of collection. Based on results, antioxidant capacity detected by DPPH and ABTS tests showed the IC_50_ values of 40–94 *µ*g/mL and 28–72 *µ*g/mL, respectively. Results of the FRAP test represented very strong ferric ion reducing activities (0.04–0.83 mmol Fe2+/g). Ferric ion reducing data were not markedly different from ABTS and DPPH ones. These samples also presented the highest phenolic content (1.32–2.28 mg GAE/100 g). Jahrom Trehala manna was the highest in both phenolic content and antioxidant value, while Sabzevar was the lowest. We found a significant relationship between the antioxidant values and total phenolic counts. It indicates that the phenolics contribute to the observed antioxidant activities of these samples.

## 1. Introduction

Manna has been used as a natural health remedy in traditional medicine. Additionally, it is being used as a mild laxative, and also, it is used in dermatological products for its antiaging and soothing activities, as an expectorant, cough suppressant, and fluidizing. The manna consumption is also considered to regulate hepatic and intestinal functions [1]. Liu et al. represented that the manna polysaccharides had the antioxidant activity [2] which is produced in plants bark, limb, and leaf either naturally or through insect's activity [3]. The most important manna types in Iranian traditional medicine are manna from willow, *Tamarisk*, *Astragalus arbusculinus*, *Cotoneaster*, *Hedysarum* manna, and Trehala manna [3]. Trehala manna is also produced in *Echinops* sp. by *Larinus* insects (Curculionidae family) [4, 5]. *Echinops* genus has about 120 species throughout the world, 54 of which have already been identified in Iran; however, Trehala manna production has only been documented in *E. dichrous*, *E. endotrichus*, *E. persepolitanus*, and *E. tenuisectus* [6]. Karam and coworkers confirmed in a previous study that the structure of water-soluble polysaccharides of Trehala manna was elucidated [7]. These manna showed at least nine distinct polysaccharides with different molecular weights, mainly containing glucose, arabinose, galactose, and xylose. In this study, it has been reported that Trehala manna has greater antioxidant activity than ascorbic acid and vitamin E [7].

According to the following studies, the antioxidant effects of manna could have some biological activity. In the recent survey carried out by Alessandro Attanzio and coworkers, the total antioxidant activity of manna extract from Sicilian *Fraxinus angustifolia* was measured by FRAP, Folin-Ciocalteu reaction, DPPH, ABTS, and radicals from perferryl-myoglobin. These findings demonstrated that the samples reduced the oxidant compounds with different potentials. In cellular models, manna extract neutralized membrane lipid oxidation of human erythrocytes and prevented ROS production, like GSH decay in the intestinal normal-like cells, in which interleukin-1*β* activates. However, in the in vitro intestinal bowel disease model, manna extract decreased the proinflammatory cytokines, interleukin 6 and interleukin 8 [1]. In another study, Azadeh Hamedi and et al. reported that the immunomodulatory effects of *Trehala* aqueous extract and its isolated polysaccharides were evaluated. Based on the results, these compounds displayed some immunomodulatory activities on the Jurkat cell line dose-dependently. The molecular weight of the polysaccharides greatly impacted their cytotoxic/proliferative properties [7]. These data suggest that manna could be both an antioxidant as well as a natural anti-inflammatory product, in addition to its benefits in the treatment of constipation.

In this study, a comprehensive investigation was performed to determine the antioxidant activity, phenolic content, flavonoid, anthocyanin, and carotenoid content of Trehala manna collected from the endemic *Echinops* species of Iran for the first time.

## 2. Materials and Methods

### 2.1. Materials

We obtained 2, 2-diphenyl-1-picrylhydrazyl (DPPH) from Sigma-Aldrich^®^. Folin–Ciocalteu, ABTS, and FRAP reagents were purchased from Merck^®^, Germany. Methanol, acetone, water (HPLC grade), and other lab chemicals or reagents were purchased from Sigma-Aldrich^®^ (Saint Louis, USA).

### 2.2. Preparation of Manna Material

Trehala manna is some type of manna exudates produced after feeding activities of insects like *Larinus* species (Curculionidae family) on *Echinops* species. Trehala manna were collected in September 2017 from sugary exudates crystalized on different *Echinops* species, including *E. macrophyllus* (voucher: 3509), *E. orientalis* (voucher: 3351), *E. ritrodes* (voucher: 3556), and *E. leiopolycresa* (Voucher: 2105), growing in Shiraz, Jahrom, Banarouyeh, Tehran, Sabzevar, and Mehriz in Iran. Mustafa Ghanadian, Pharmacognosy professor, identified *Trahala manna*, and voucher specimens were stored in Samsam-Shariat Herbarium, Isfahan Pharmacognosy Department, Isfahan University of Medical Sciences, Iran.

After collection, the samples were cleaned from insect residuals and then milled by a grinder and sieved with a 60-mesh size to obtain uniform particles. The grinded samples of Trehala manna were stored in air-tight plastic bags in the dark under refrigeration conditions until further use.

For the determination of phenolic compounds by HPLC analysis, samples were prepared freshly before use. Briefly, the weighed solid samples of each type of Trehala manna (400 mg) were added to 4 mL of ethanol-water (1 : 1) containing 0.1% acetic acid. The resulting mixtures were sonicated twice and kept at 70°C for 10 minutes. It was filtered and kept in the refrigerator until use. For other assays including antioxidant, total phenolic, and total flavonoid contents, the extraction processes were explained according to the method, separately.

### 2.3. ABST Radical Scavenging Assay

Antioxidant activities of Trehala manna samples were investigated according to the method presented by Re et al. We prepared cation radical of ABTS^+^ through the reaction of ABTS^+^ in water (7 mM) and potassium persulfate (2.45 mM) in the ratio of 1 : 1. It was kept in the dark condition for 16 hours (room temperature) before use. The ABTS^+^ radical cation was diluted with pure ethanol to reach the absorption of 0.7 at 734 nm. After that, 2 mL of ABTS was mixed with 2 mL of sample extracts and was then stirred. ABTS solution was used as the control. Samples were left for 10 minutes at room temperature; then, the absorptions of these samples were measured at 734 nm. We read the absorbance 10 min after initial mixing at 30°C. Solution preparations and all determinations were performed in triplicate. The following equation obtained the inhibitory percentage of ABTS+•:

(1) $$\begin{array}{c} {\text{ABTS}}^{+}\text{scavenging percentage}=\frac{\;A_{\text{control}}-\;A_{\text{sample}}}{\;A_{\text{control}}}\times 100. \end{array}$$

*A*_sample_ is the absorbance of the sample after 6 minutes, and *A*_control_ is the absorbance of the control after 10 minutes.

### 2.4. DPPH Free Radical Scavenging Assay

This assay was performed according to the procedure described by Wang et al. [8]. Briefly, 50 *µ*L of aqueous Trehala manna samples at different concentrations (50–300 *µ*g/mL) were added in 50 *µ*L of a DPPH solution (0.1 mM in ethanol). Ascorbic acid was employed as a positive control. The obtained mixture was vigorously mixed on a shaker and stored for 30 minutes at 37°C in the dark. The absorbance of the solutions was measured at 517 nm on a UV-visible spectrophotometer (Genesis, USA). DPPH free radical scavenging (FRS) activity was calculated by using the following equation:

(2) $$\begin{array}{c} \text{scavenging ability}\left(\%\right)=\frac{1\;-\;A_{\text{sample}}\;}{A_{\text{control}}\;}\times 100. \end{array}$$

*A*_sample_ is the absorbance of the solutions containing different Trehala manna concentrations, and *A*_control_ is the absorbance at 517 nm of the solution without Trehala manna.

### 2.5. Measurement of Ferric Ion Reducing Power (FRAP)

According to the procedure of the ferric ion reducing power (FRAP) [9, 10], the FRAP reagent was freshly made from a 1 : 1 : 10 mixture of solutions: ferrous chloride (20 mM), 2,4,6-tripyridyl-S-triazine (TPTZ) (10 mM), and acetate buffer (0.3 M, pH 3.6). Then, 100 *µ*L of diluted Trehala manna sample was mixed with 4.1 mL of FRAP solution, and its absorbance was read at 593 nm.

### 2.6. Evaluation of Total Phenol Content

The total phenol content of Trehala manna samples was measured by Folin–Ciocalteu reagent according to the modified version of the method described by Singleton and Rossi (1965) [11]. Briefly, 0.5 mL of manna samples (0.1 mg/mL) was mixed with 0.5 mL Folin–Ciocalteu reagent. The obtained mixtures were left for 5 minutes at room temperature. Then, 0.5 mL of 20% (w/v) sodium carbonate solution was added to the mixtures and then stirred gently. The mixtures were diluted to a final volume of 5 mL with distilled water. Then, they were homogenized and stored at room temperature in the dark for 30 minutes, after which the absorbance of the solutions was measured at 725 nm, and their total phenol content was calculated using a standard curve using gallic acid (0–300 mg/L). The total phenol content of Trehala manna samples was calculated based on gallic acid equivalents (GAE) and of mg/100 g of Trehala manna sample [11].

### 2.7. Evaluation of Total Flavonoid Content

**Figure 1:**
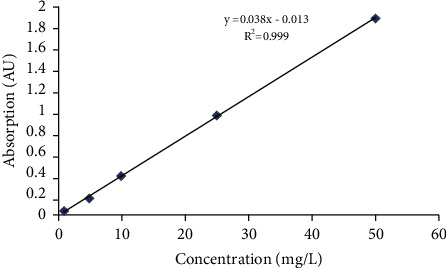
Standard curve of quercetin for the determination of total flavonoid content in different Trehala manna samples.

Total flavonoid contents in Trehala manna samples were determined using UV spectrophotometry. 1 mL (1 mg/mL methanol) of different Trehala manna samples was mixed with 1 mL aluminum chloride (2% in methanol). The samples were incubated at room temperature for 1 h. The absorption of samples was measured at 415 nm in three replicates. A similar method was used for quercetin standard solutions, and a standard curve was drawn (Figure 1). Finally, total flavonoid contents were calculated and reported as quercetin equivalent (QUE) (mg/QUE of the sample) [12].

### 2.8. Evaluation of Total Anthocyanin Content

Total anthocyanin contents in Trehala manna samples were determined according to Giusti and Wrolstad's method with minor modifications. 100 mg of each sample was dissolved in 100 mL distilled water and used for measuring anthocyanin concentration. Absorptions of the samples were measured using visible spectrophotometry (Genesis, USA) at wavelengths of 510 nm and 700 nm in pH of 1.0 and 4.5 buffer solutions, respectively. Total anthocyanin contents were determined using cyanidin-3-glucoside molar absorption coefficient (*ε* = 269001) as follows:

(3) $$\begin{array}{c} A=\left[\left(A510-A700\right)\text{pH}1.0-\left(A510-A700\right)\text{pH}4.5\right]. \end{array}$$

### 2.9. Measurement of Total Carotenoid Content

Extracts of different manna samples of Trehala manna were mixed with hexane-ethanol (with ratio 9 : 1 by volume) and centrifuged at 1000 rpm for 5 minutes. After filtration, the absorbance of the samples was measured at 625 nm, and total carotenoid contents were calculated according to the following equation:

(4) $$\begin{array}{c} \;\frac{A*V*10^{6}}{2500*100*g}=\text{total carotenoid }\left(\frac{mg}{g}\right), \end{array}$$

where *V* represents the final volume, *A* represents the maximum absorption, and *g* represents the sample weight in g.

### 2.10. Determination of Phenolic Compounds Present in Trehala Manna Samples Using HPLC

**Figure 2:**
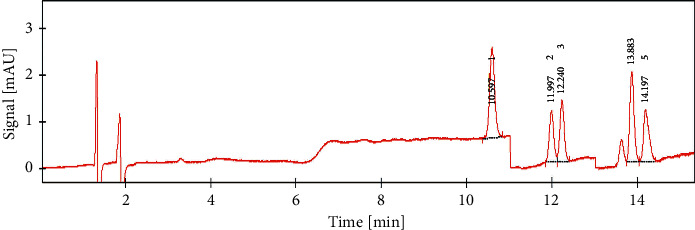
HPLC profile of phenolic acid standards. 1, parahydroxybenzoic acid (rt = 10.6 min); 2, caffeic acid (rt = 12.0 min); 3, syringic acid (rt = 12.2 min); 4, coumaric acid (rt = 13.9 min); and 5, ferulic acid (rt = 14.2 min).

Phenolic compounds present in different Trehala manna samples were determined using a modified version of the Wen et al. (2005) method. Manna samples (400 mg) were added to 4 mL of ethanol-water (1 : 1) containing 0.1% acetic acid, and the resulting mixtures were sonicated twice and kept at 70°C for 10 minutes. Finally, the extracts were injected into HPLC apparatus (HPLC Rigol L-300) equipped with a UV-Vis detector using an RP-18 (150 mm, 4.6 mm, with mesh size 5 *µ*m) HPLC column (Merck, Germany) (Figure 2).

### 2.11. Statistical Analysis

Using SPSS software, the meaningfulness of treatment was evaluated using a one-way analysis of variance in the Kolmogorov–Smirnov test. The Duncan test was used to compare the average values in cases where the total effect of treatment was found meaningful.

## 3. Results

### 3.1. ABST Radical Scavenging Assay

Generally, common phenolic compounds possessing antioxidant activity are phenolic acids and flavonoids. Flavonoids based on their chemical structure are classified mainly into flavonols, flavones, flavanones, isoflavones, catechins, and anthocyanidins. Phenolic acids are a major group of phenolic compounds extensively found in medicinal plants, especially fruits and vegetables. Total phenolic content alone is not a precise and constant criterion for the confirmation of the high antioxidant power of a sample, but the nature, type, and concentration of its individual compounds are important. TEAC assay checks the potential of samples to scavenge 2, 2′-azino-bis (3-ethylbenzothiazoline-6-sulphonic acid), which is a stable radical cation with maximum absorption at 734 nm in which its OD is reduced antioxidant samples. Based on this method, the total antioxidant capacities of Trehala manna samples are given in Table 1. These data showed that the Trehala manna samples obtained from the Jahrom region had the highest TEAC potential among all the samples. Furthermore, Table 2 shows that there is a meaningful difference in phenolic acid contents from different Trehala manna samples (*p* < 0.05), which may be due to environmental conditions such as climate, region, temperature, fertility, diseases, and exposure to pests [13–15].

### 3.2. DPPH Free Radical Scavenging Assay

In this method, pink stable radical DPPH (*λ*max = 517 nm) is neutralized to a colorless compound by proton donation or radical scavenging ability. According to the obtained results, it was found (Table 1) that the effect of the plant gathering region on the free radical inhibition activity of DPPH was meaningful at a 5% level. Free radical inhibition activity of DPPH in different Trehala manna samples ranged from 40% to 94%. Jahrom manna had the lowest free radical inhibition activity for DPPH. Tehran, Banarouyeh, Mehriz, Shiraz, and Sabzevar were ranked in DPPH values in the increasing order. By the before said mechanism, the polysaccharides in different Trehala manna samples could act as electron donors and react with DPPH free radicals and consequently transform them into stable products. These results are in agreement with the investigation of polysaccharide compounds in *Helicteres angustifolia* plants which showed the free radical inhibition activity of 32.81%–89.83% [16]. Liu et al. (2017) also performed three tests, including detection of free radical of superoxide anion, free radical test, and detection of DPPH free radical on *Schisandra sphenanthera* plant, which is rich in polysaccharides and found a direct relationship between the concentration of water-soluble polysaccharides and antioxidant activity [17]. According to the obtained results, it was observed that the effect of region on DPPH free radical inhibition was meaningful at a 5% level. Manna obtained from Trehala manna collected from Jahrom (IC_50_% = 39.705 ± 1.322 *µ*l) had the highest DPPH free radical inhibition capacity. The samples collected from Tehran, Banarouyeh, Mehriz, Shiraz, and Sabzevar were in the following rankings. DPPH free radical inhibition activity among Trehala manna samples ranged 39.70%–93.72%. Salmanian et al. [18] reported that by increasing the percentage of the extract, its inhibition capacity was increased [19], which suggests a direct relation between reducing property and phenolic compound percentage. Free radicals can destroy different macromolecules like proteins, nucleic acids, and lipids [20]. In the case of carbohydrates, free radicals break carbon-hydrogen bonds in aldose ring, uronic acid, and all carbohydrate groups except C2 in N-acetyl hexamine [21].

Natural extracts are considered valuable antioxidant sources, especially polysaccharides that have the ability of free radical tracing. Research studies have shown that some polysaccharides can inhibit lipid peroxidation, enhance free radical's inhibition ability of the body, and inhibit cardiovascular and brain diseases. According to the obtained results, the Trehala manna sample collected from Jahrom (IC_50_% = 27.703 ± 1.169 *µ*l) had the highest Trolox equivalent antioxidant potential among all samples, and the lowest value was obtained from the sample collected from Sabzevar (IC_50_% = 72.503 ± 2.734 *µ*l). Relatively stable DPPH radical is extensively being used in the determination of antioxidant activities of different individual compounds and plant extracts [22].

### 3.3. Measurement of Ferric Ion Reducing Power (FRAP)

The antioxidant properties of different Trehala manna samples are given in Table 1.

### 3.4. Evaluation of Total Phenol Content

Total phenolic content in Trehala manna was measured according to Folin–Ciocalteu. The total amount of phenols, in the range of 1.3–2.3 mg GAE per 100 g, is relatively low (only about 0.01-0.02% of the total weight of the manna samples). Also, there was quite a lot of variation in the samples. Manna obtained from Jahrom had the highest total phenolic content, and samples obtained from Mehriz, Tehran, Sabzevar, Shiraz, and Banarouyeh were in the next rankings, respectively (Table 3). It was found that the effect of the plant gathering region on the phenolic content of samples was meaningful.

### 3.5. Evaluation of Total Flavonoid Content

In this work, the flavonoid contents of the samples were determined in terms of mg quercetin/g of extract (Table 3). The obtained results showed that Trehala manna samples obtained from Shiraz and Jahrom had the highest flavonoid contents among all samples examined (8.275 ± 0.388 and 7.935 ± 0.049 mg QUE per 100 g, respectively). On the other hand, samples collected from Banarouyeh had the lowest flavonoid content (4.680 ± 0.480), and the Trehala manna sample obtained from Banarouyeh had the least concentration of this compound (0.015 ± 0.11).

### 3.6. Evaluation of Total Anthocyanin Content

Anthocyanin pigments create red, purple, and blue colors in many fruits, vegetables, cereals, and plant structures. More than 300 anthocyanin types have been identified to be antioxidant compounds due to the distinctions in their structures [23]. By investigating the concentrations of anthocyanins in different Trehala manna samples (Table 2), it was observed that samples collected from Jahrom and Shiraz had the highest concentrations of anthocyanin among all the samples (0.036 ± 0.014 and 0.033 ± 0.007 mg/g cyanidin equivalent, respectively), and the lowest concentration of this compound was found in samples collected from Banarouyeh (0.015 ± 0.011). The presence of catechin and anthocyanins may describe high antioxidant capacity in plants. It has been reported that procyanidins had the highest FRS capacity among the all-natural phenolic compounds [24].

### 3.7. Measurement of Total Carotenoid Content

According to the obtained results (Table 3), Trehala manna samples collected from Shiraz and Jahrom had the highest concentrations of carotenoid (8.0915 ± 1.025 and 7.880 ± 1.697, respectively), and its lowest concentration has been detected in the samples collected from Banarouyeh (2.590 ± 1.038). Carotenoids include *ß*-carotene, *α*-carotene, *ß*-cryptoxanthin, and *α*-cryptoxanthin, which can be transformed to vitamin A in the body [25]. They are a vital group of fat-soluble functional compounds and are extensively found in plants, animals, and microbes. Carotenoids possess several important functions including antioxidant activity, prevention of cardiovascular diseases, and cancer, and in some cases, they act as vitamin precursors [26].

### 3.8. Determination of Phenolic Compounds Present in Trehala Manna Samples Using HPLC

As indicated in Table 2, the plant gathering region has a significant effect on the content of phenolic acids in Trehala manna samples (*p* < 0.05). The contents of hydroxybenzoic acid and syringic acid were similar in all treatments and lower than 0.1 and 0.2 mg/kg, respectively. The Trehala manna sample collected from Jahrom has the highest percentages of caffeic (3.2 mg/kg) and coumaric (1.9 mg/kg) acids. Samples were collected from Shiraz with 2.5 mg/kg caffeic acid and 1.87 mg/kg coumaric acid in the second ranking order. Trehala manna samples collected from Tehran had the highest ferulic acid content among all five samples, and that collected from Jahrom had the lowest amount.

## 4. Discussion

The antioxidant potential of herbal extracts is not only related to the extracted ingredient but also to the conditions of the assay used. The numerous published methods for determining the total antioxidant capacity have been reported in the in vitro model, which can be categorized in two types: hydrogen atom transfer assays (HAT) and electron transfer assays (ET). HAT assays exert a competitive reaction plot, in which substrate and antioxidant compete for thermally producing peroxyl radicals. ET assays quantify the antioxidant capacity to diminish an oxidant activity, which convert the color when reduced. The grade of color converting is associated with the concentration of the antioxidant sample. ET assays comprise the total phenols assay by ABTS, DPPH radical scavenging capacity assays, Folin–Ciocalteu reagent, and the FRAP assay [27]. No single assay is adequate; more than one type of procedure to evaluate the antioxidant capacity must be accomplished to consider the different mechanisms of action of antioxidants [27, 28]. In the present study, the FR's capacities of the selected manna were measured by DPPH and ABTS assays, and the FRAP assay determined their ferric reducing capacities. ABTS, FRAP, and DPPH assays are used to measure the antioxidant powers of extract and compounds as they need comparatively standard implementation and deliver expeditious and reproducible outcomes. Undoubtedly, these four methods for determining the antioxidant capacities represented that ABTS and DPPH assays are the simplest with the most reproducible results [29]. One of the advantages of the ABTS is the wavelength absorption at 734 nm, which eliminates color interference [30].

These assays, such as FRAP, DPPH, and ABTS, represented comparable outcomes for the antioxidant activity determined in aqueous extracts of six selected manna. In the study, has confirmed by Thaipong et al., it was reported that the most correlations were detected between DPPH and ABTS assays [31], and **t**he lowest correlations were detected between the FRAP assay and others.

Remarkable correlations were also detected between DPPH, ABTS, and the amount of phenolic acid (caffeic acid and coumaric acid) content determined by the HPLC method. These results indicate a relationship between phenolic acid content in manna and their FRS and ferric reducing capacities. Hence, the presence of phenolic acid components in manna contributes remarkably to their antioxidant capacity. These findings are in accordance with previous reports that FR's potential can be related to phenolic content [32, 33]. Antioxidant characteristics of phenolic compounds are directly associated with their chemical structures. Phenolic compounds consist of one or more than one aromatic rings with at least one OH group. They can conceivably quench the produced free radicals via generating resonance-stabilized phenoxyl radicals [34, 35].

Through the six selected manna were analysed, Sabzevar and Shiraz possessed the most antioxidant characteristics. In a recent study on manna of some *Fraxinus* species, the anti-inflammatory and antioxidant capacities of these phytocomplex compounds were reported [1]. Manna is an important potential source of polysaccharides and natural antioxidants [1, 7]. Here, it was detected Sabzevar manna possessed high antioxidant properties by DPPH and ABTS assays. It might be because of a high polysaccharide and macromolecule carbohydrate content as well as high anthocyanin content, and Shiraz by FRAP assay that might probably be associated with a very high polysaccharide and macromolecule carbohydrate content and also highest flavonoid and carotenoid content [7] more than that of several commercial antioxidant-rich plants and well-known antioxidant-plant extracts [31]. The study that Bertrand Payet and coworkers have confirmed represented that the brown sugar extracts exhibited interesting FRS activities despite the low content of phenolic compounds [36]. It could be related to the antioxidant activity of sugars such as fructose, glucose, and ribose, and polysaccharides that correlate the effectiveness of different Maillard reaction products to scavenge free radicals substrates and reaction conditions [37, 38]. On the other side, Jahrom manna showed the highest phenolic compound comprising the highest caffeic and coumaric acid content. However, it had the lowest antioxidant properties by DPPH and ABTS assays that it is probably related to the content of the sugars and polysaccharides. In the present study, the prosperous source of natural antioxidants was identified from Trehala manna that was poorly studied and represented good antioxidant capacities. Additional investigations are required to identify Trehala manna extracts' active components and biological effects to be included in supplements or nutraceutical formulations.

## 5. Conclusions

For the first time, this work reports evidence regarding the Trehala manna's antioxidant properties and phytochemical content, which may prepare a scientific proof of this product's traditionally recorded health benefits.

In the present study, the polyphenol composition of an aqueous extract of manna was reported, and its antioxidant capacity was demonstrated.

The high levels of phenols/polyphenols in our extracts, with caffeic acid, coumaric acid, and ferulic acid as the major components, and the high content of flavonoid, carotenoid anthocyanin, and saccharide/polysaccharide can account for the FRS properties of manna.

Well-known manna health benefits have frequently been described and are in accordance with the characteristics of mannitol, a polyol with osmotically active cell-compatible [39]. The antioxidative effects here determined extrapotential functional features to this product. Despite the wide experimental approaches confirmed in this study, aiming to assay the antioxidative potential of different collections of Trehala manna, a limitation of the present study is related to the in vitro system. In this way and considering the current outcomes suggesting the potential Trehala manna to control redox-regulated signaling, investigation on the effects of Trehala manna in normal and pathological in vivo approaches are in progress.

## Figures and Tables

**Table 1:** Antioxidant properties of different Trehala manna samples.

Sample (region)	Inhibition activity, DPPH^•^ (IC_50_%)	Inhibition activity, ABTS^+•^ (IC_50_%)	FRAP test (mmol Fe^2+^/g)
Shiraz	81.00 ± 1.32	64.00 ± 2.90^a^	0.830 ± 0.014^a^
Jahrom	39.71 ± 1.32^d^	27.70 ± 2.90^b^	0.153 ± 0.014^b^
Banarouyeh	63.37 ± 1.32^c^	63.87 ± 2.90^a^	0.106 ± 0.014^c^
Tehran	42.85 ± 1.32^d^	34.65 ± 2.90^b^	0.155 ± 0.014^b^
Sabzevar	93.72 ± 1.32^a^	72.50 ± 2.90^a^	0.069 ± 0.014^cd^
Mehriz	63.87 ± 1.32^c^	39.06 ± 2.90^b^	0.045 ± 0.014^d^

*a*, *b*, *c*, and *d* in each column indicate significant differences between treatments at *p* < 0.05.

**Table 2:** Phenolic acids contents in different Trehala manna samples.

Phenolic acids (mg/kg)					
Sample	Hydroxybenzoic acid	Caffeic acid	Syringic acid	Coumaric acid	Ferulic acid
Shiraz	n.d.	2.5	n.d.	1.87	5.7 × 10^−1^
Jahrom	n.d.	3.2	n.d.	1.9	1.3 × 10^−1^
Banarouyeh	n.d.	1.2	n.d.	1.6	5.1 × 10^−1^
Tehran	n.d.	2.1	n.d.	1.7	8.2 × 10^−1^
Sabzevar	n.d.	1.5	n.d.	1.8	6.7 × 10^−1^
Mehriz	n.d.	1.4	n.d.	1.7	7.1 × 10^−1^

**Table 3:** Total phenolic compounds, flavonoid, carotenoid, and anthocyanin in Trehala manna samples collected from different regions.

Sample	Phenolic compounds (mg GAE/100 g)	Flavonoids (mg QUE/100 g)	Total carotenoid (mg/g)	Anthocyanins (mg cyanidin/g)
Shiraz	14.0 ± 2.1^c^	8.28 ± 0.73^a^	8.09 ± 1.13^a^	0.025 ± 0.005^b^
Jahrom	23.0 ± 2.1^a^	7.94 ± 0.73^a^	7.88 ± 1.13^ab^	0.033 ± 0.005^a^
Banarouyeh	13.0 ± 2.1^d^	4.68 ± 0.73^b^	2.59 ± 1.13^c^	0.015 ± 0.005^c^
Tehran	20.0 ± 2.1^b^	7.12 ± 0.73^a^	4.40 ± 1.13^bc^	0.019 ± 0.005^bc^
Sabzevar	15.0 ± 2.1^c^	7.12 ± 0.73^a^	4.77 ± 1.13^bc^	0.036 ± 0.005^a^
Mehriz	21.0 ± 2.1^b^	4.87 ± 0.73^b^	3.08 ± 1.13^c^	0.017 ± 0.005^bc^

*a*, *b*, *c*, and *d* in each column indicate significant differences between treatments at *p* < 0.05.

## Data Availability

The data used to support the findings of this study are available from the corresponding author upon request.

## Abbreviations

ABTS: 2,2′-Azino-bis(3-ethylbenzothiazoline-6-sulphonic acid)
DPPH: 2, 2-Diphenyl-1-picrylhydrazyl
HPLC: High-performance liquid chromatography
FRAP: Ferric ion reducing power
GAE: Gallic acid
IL: Interleukin
TEAC: Trolox equivalent antioxidant capacity
OD: Optical density
IC: Inhibitory concentration
QUE: Quercetin.
